# Detection of antibodies to human herpesvirus 8 in Italian children: evidence for horizontal transmission

**DOI:** 10.1054/bjoc.1999.0983

**Published:** 2000-01-18

**Authors:** D Whitby, M Luppi, C Sabin, P Barozzi, A R Di Biase, F Balli, F Cucci, R A Weiss, C Boshoff, G Torelli

**Affiliations:** Institute of Cancer Research, London, UK; Departments of Medical SciencesPaediatrics, Section of Haematology, University of Modena, Policlinico, Italy; Department of Primary Care and Population Sciences, Royal Free and University College Medical School, London, UK; Blood Transfusion Center, Brindisi, Italy

## Abstract

Human herpesvirus 8 (HHV-8), also known as Kaposi's sarcoma associated herpesvirus (KSHV), has been shown to be the causative agent for Kaposi's sarcoma (KS) and to be more prevalent in populations or risk groups at increased risk for KS. HHV-8 infection is rare in children from the US and the UK, but has been reported in African children. In this study we examine HHV-8 infection in children from Italy, a country with an elevated prevalence of HHV-8 in adults and high socio-economic conditions. © 2000 Cancer Research Campaign


British Journal of Cancer (2000) **82**(3), 702–704
© 2000 Cancer Research Campaign
DOI: 10.1054/ bjoc.1999.0983, available online at http://www.idealibrary.com on IDEAL®

Short Communication

### Detection of antibodies to human herpesvirus 8 in Italian children: evidence for horizontal transmission

**D Whitby[1]*, M Luppi[2], C Sabin[4], P Barozzi[2], AR Di Biase[3], F Balli[3], F Cucci[5], RA Weiss[1†], C Boshoff[1†] and G Torelli[2]**

[1]Institute of Cancer Research, London, UK; Departments of [2]Medical Sciences and [3]Paediatrics, Section of Haematology, University of Modena, Policlinico, Italy; [4]Department of Primary Care and Population Sciences, Royal Free and University College Medical School, London, UK; [5]Blood Transfusion Center, Brindisi, Italy

**Summary** Human herpesvirus 8 (HHV-8), also known as Kaposi's sarcoma associated herpesvirus (KSHV), has been shown to be the causative agent for Kaposi's sarcoma (KS) and to be more prevalent in populations or risk groups at increased risk for KS. HHV-8 infection is rare in children from the US and the UK, but has been reported in African children. In this study we examine HHV-8 infection in children from Italy, a country with an elevated prevalence of HHV-8 in adults and high socio-economic conditions. © 2000 Cancer Research Campaign

Serological and polymerase chain reaction (PCR)-based studies have shown that human herpesvirus-8 (HHV-8) infection is infrequent in the general population of the UK and the USA, but common in groups at increased risk for Kaposi's sarcoma (KS) such as HIV-infected homosexual men (Gao et al, 1996; Kedes et al, 1996; Lennette et al, 1996; Simpson et al, 1996). Antibodies to HHV-8 are detected more frequently in sexually transmitted disease (STD) clinic attendees than in blood donors, suggesting that HHV-8 may be sexually transmitted (Kedes et al, 1996; Simpson et al, 1996). Direct evidence for homosexual transmission of HHV-8 has been reported (Martin et al, 1998; Melbye et al, 1998; Renwick et al, 1998; Smith et al, 1999). Evidence for transmission via heterosexual contact, however, is less consistent. A large study of risk factors for HHV-8 infection in South African cancer patients found that the risk of HHV-8 infection was associated with increased numbers of sexual partners (Sitas et al, 1999). A study of STD clinic attendees in London, however, found no evidence for sexual transmission in heterosexuals, including those born in Africa (Smith et al, 1999). HHV-8 infection does not occur in children in the UK (Simpson et al, 1996), and in the US occurs rarely, after puberty (Blauvelt et al, 1997), as would be expected for a sexually transmitted virus.

HHV-8 infection has been shown to be more common in adult blood donors from geographical areas which have an elevated incidence of classic KS, such as Italy and Greece (Calabro et al, 1998; Rezza et al, 1998; Whitby and Boshoff, 1998; Whitby et al, 1998), or African endemic KS (Gao et al, 1996; Lennette et al, 1996; Simpson et al, 1996; Mayama et al, 1998; Olsen et al, 1998). In Italy, where marked regional variation occurs in the incidence of KS (Geddes et al, 1994, 1995), HHV-8 prevalence has been shown to mirror KS incidence, being significantly higher in the south than the north (Calabro et al, 1998; Whitby et al, 1998). KS occurs in children in Africa (Ziegler and Katongole-Mbidde, 1996) and HHV-8 infection in African children has been reported (Kasolo et al, 1997; Bourboulia et al, 1998; Mayama et al, 1998; Andreoni et al, 1999). One report provided evidence that HHV-8 infection in African children may be transmitted from mother to child (Bourboulia et al, 1998). Prevalence of HHV-8 in African children increases with age even before puberty (Bourboulia et al, 1998; Mayama et al, 1998; Andreoni et al, 1999), indicating that non-sexual horizontal transmission of the virus occurs. Detection of antibodies to HHV-8 in a small study of Ugandan children suggested an association between HHV-8 and hepatitis B infection, indicating that transmission of HHV-8 in childhood is associated with living conditions which may also facilitate infection with hepatitis B virus (Mayama et al, 1998). Childhood KS does not occur in regions with a high incidence of classic KS such as Italy and Greece. The prevalence of HHV-8 in children from non-African regions with a high incidence of classic KS and a high prevalence of HHV-8 in the adult population, is not known. Here we report on the prevalence of HHV-8 in children in Italy.

### MATERIALS AND METHODS

We investigated the prevalence of antibodies to HHV-8 in 567 hospitalized children (306 male, 261 female) from three different regions of Italy, including 388 children from Modena, Emilia-Romagna (lower Po valley, Northern Italy), 76 from Brindisi and Bari, Apulia (Southeastern Italy) and 103 children from Palermo, Sicily (Southern Italy). Sera were collected from consecutive children admitted to the Departments of Pediatrics at each site. The vast majority were admitted for a single day and were being treated for minor childhood complaints. All children included in the study were seronegative for HIV-1, hepatitis B virus (HBV), HCV and *Treponema pallidum*, and did not have KS. Antibodies

*Received 7 April 1999*
*Revised 18 May 1999*
*Accepted 18 May 1999*

*Correspondence to:* D Whitby, National Cancer Institute–Frederick Cancer Research and Development Center, PO Box B, Frederick, MD21702-1201 USA

*Present address: National Cancer Institute–Frederick Cancer Research and Development Center, PO Box B, Frederick, MD21702-1201 USA

†Present address: Departments of Oncology and Molecular Pathology, University College London, UK

**Table 1** Prevalence of antibodies against HHV-8 LNA-1 in children and adult blood donors from Italian regions

| Region (city) | Children | | | | Adult blood donors |
|---|---|---|---|---|---|
| | No. tested | No. positive | % HHV-8 positive | 95% CI | % HHV-8 positive[a] |
| Emilia Romagna (Modena) | 388 | 16 | 4.1 | 2.4–6.6 | 12.7 |
| Apulia (Brindisi, Bari) | 76 | 3 | 3.9 | 0.8–11.1 | 24.2 |
| Sicily (Palermo) | 103 | 6 | 5.8 | 2.2–12.2 | 35 |
| Overall | 567 | 25 | 4.4 | | |

[a]Data previously reported in Whitby et al, *J Natl Cancer Inst*, 1998, **90**, 395–397

**Table 2** Age-dependent seroprevalence rates for HHV-8 in children from Emilia Romagna (Modena)

| Age (years) | Total No. | Positive No. | Frequency % | 95% CI |
|---|---|---|---|---|
| <1 | 57 | 2 | 3.5 | 0.4–12.1 |
| 1–2 | 109 | 4 | 3.6 | 1.0–9.1 |
| 3–5 | 90 | 1 | 1.1 | 0.0–6.0 |
| 6–10 | 94 | 6 | 6.3 | 2.4–13.4 |
| 11–15 | 38 | 3 | 7.8 | 1.7–21.4 |

to the HHV-8 latent nuclear antigen (LNA-1) were detected by an immunofluorescence assay (IFA) using a HHV-8 infected peripheral effusion lymphoma (PEL) cell line, BCP-1 (Gao et al, 1996; Boshoff et al, 1998). LNA-1 IFA is the most sensitive and specific assay currently available to reveal past or present infection with HHV-8 (Rabkin et al, 1998). Sera were tested in a blinded manner at a dilution of 1:100, as previously described (Simpson et al, 1996; Whitby et al, 1998).

### RESULTS

The results of the serological survey are summarized in Table 1. The prevalence of antibodies against HHV-8 LNA-1 in Italian children was 4.4%. HHV-8 seroprevalence was 4.1% in Northern Italy (the Po valley) and 5% in Southern Italy (Apulia and Sicily), which is not significantly different ($P = 0.66$, Fisher's exact test). There was no significant difference in HHV-8 antibody prevalence between males and females (14/306, 4.6%, males; 11/261, 4.2% females, $P = 1.00$ $\chi^2$ test). Information on age was available for children from the Po valley (Table 2). Of 57 infants (< 1 year), two (3.5%) had antibodies to HHV-8 (6 and 9 months old respectively). It is possible that maternal antibodies to HHV-8 were detected in these infants. A similar seroprevalence rate (3.6%) was detected in children aged 1 and 2 years. Antibodies to HHV-8 were detected in only one of 90 children aged 3–5 years (1.1%). Seroprevalence rates increased in children aged 6–10 years (6.3%) and 11–15 years (7.8%) respectively. Children aged 6 and over have a 160% increased chance of being positive compared to younger children (odds ratio 2.60, 95% confidence interval (CI) 0.95–7.5, $P = 0.06$).

### DISCUSSION

The results of our study show that HHV-8 infection occurs in Italian children before puberty suggesting that non-sexual routes of transmission of HHV-8 are important in a country with a relatively high seroprevalence rate in the adult population. Vaccination to HBV was introduced into Italy several years ago. It is therefore not possible to investigate in this study whether factors facilitating HBV transmission also play a role in the transmission of HHV-8 as has been suggested in Uganda (Mayama et al, 1998). However, crowded living conditions and poor hygiene associated with HBV transmission in Africa are not found in Italy and therefore may not play a major role in HHV-8 transmission.

While in adult blood donors the presence of antibodies to HHV-8 is significantly higher in Southern than in North/Central Italy (Calabro et al, 1998; Whitby et al, 1998), in children HHV-8 seroprevalence rates show no differences between the regions surveyed. The numbers studied were low, however, so it is possible that real differences exist and could be demonstrated in a larger study. Whilst seroprevalence increases with age in children from the Po valley, especially in those aged 6–10 years, it remains lower than in adults. In contrast, seroprevalence in Ugandan children is reported to reach adult levels before puberty (Mayama et al, 1998). Overall, these findings suggest that risk factors for HHV-8 transmission differ between children and adults and that in Italy, marked regional differences occur in the risk factors affecting adults more than children.

Clustering of HHV-8 seroprevalence rates have been documented in spouses, children and siblings of patients with classic KS from Sardinia, Italy, suggesting the occurrence of intrafamilial, horizontal or vertical transmission (Angeloni et al, 1998). Further studies are needed to determine the routes of transmission of HHV-8 in children. HHV-8 has been detected in the saliva of HIV-infected KS patients (Koelle et al, 1997; Vieira et al, 1997), indicating one possible source of virus; however, studies in immunocompetent HHV-8 positive individuals have yet to be reported.

### ACKNOWLEDGEMENTS

This work was funded by the Medical Research Council, the Cancer Research Campaign and the Associazione Italiana per la Ricerca sul Cancro.

### REFERENCES

Andreoni M, El-Sawaf G, Rezza G, Ensoli B, Nicastri E, Ventura L, Ercoli L, Sarmati L and Rocchi G (1999) High seroprevalence of antibodies to human herpesvirus-8 in Egyptian children: evidence of nonsexual transmission. *J Natl Cancer Inst* **91**: 465–469

Angeloni A, Heston L, Uccini S, Sirianni MC, Cottoni F, Masala MV, Cerimele D, Lin SF, Sun R, Rigsby M, Faggioni A and Miller G (1998) High prevalence of

*© 2000 Cancer Research Campaign* 

antibodies to human herpesvirus 8 in relatives of patients with classic Kaposi's sarcoma from Sardinia. *J Infect Dis* **177**: 1715–1718

Blauvelt A, Sei S, Cook PM, Schulz TF and Jeang KT (1997) Human herpesvirus 8 infection occurs following adolescence in the United States. *J Infect Dis* **176**: 771–774

Boshoff C, Gao SJ, Healy LE, Matthews S, Thomas AJ, Coignet L, Warnke RA, Strauchen JA, Matutes E, Kamel OW, Moore PS, Weiss RA and Chang Y (1998) Establishing a KSHV+ cell line (BCP-1) from peripheral blood and characterizing its growth in Nod/SCID mice. *Blood* **91**: 1671–1679

Bourboulia D, Whitby D, Boshoff C, Newton R, Beral V, Carrara H, Lane A and Sitas F (1998) Serologic evidence for mother-to-child transmission of Kaposi sarcoma-associated herpesvirus infection. *Jama* **280**: 31–32

Calabro M, Sheldon J, Favero A, Simpson G, Fiore J, Gomes E, Angarano G, Chieco-Bianchi L and Schulz T (1998) Seroprevalence of Kaposi sarcoma-associated herpesvirus/human herpesvirus 8 in several regions of Italy. *Journal of Human Virology* **1**: 207–213

Gao SJ, Kingsley L, Li M, Zheng W, Parravicini C, Ziegler J, Newton R, Rinaldo CR, Saah A, Phair J, Detels R, Chang Y and Moore PS (1996) KSHV antibodies among Americans, Italians and Ugandans with and without Kaposi's sarcoma. *Nat Med* **2**: 925–928

Geddes M, Franceschi S, Barchielli A, Falcini F, Carli S, Cocconi G, Conti E, Crosignani P, Gafa L, Giarelli L and et al. (1994) Kaposi's sarcoma in Italy before and after the AIDS epidemic. *Br J Cancer* **69**: 333–336

Geddes M, Franceschi S, Balzi D, Arniani S, Gafa L and Zanetti R (1995) Birthplace and classic Kaposi's sarcoma in Italy. Associazione Italiana Registri Tumori. *J Natl Cancer Inst* **87**: 1015–1017

Kasolo FC, Mpabalwani E and Gompels UA (1997) Infection with AIDS-related herpesviruses in human immunodeficiency virus-negative infants and endemic childhood Kaposi's sarcoma in Africa. *J Gen Virol* **78**: 847–855

Kedes DH, Operskalski E, Busch M, Kohn R, Flood J and Ganem D (1996) The seroepidemiology of human herpesvirus 8 (Kaposi's sarcoma-associated herpesvirus): distribution of infection in KS risk groups and evidence for sexual transmission. *Nat Med* **2**: 918–924

Koelle DM, Huang ML, Chandran B, Vieira J, Piepkorn M and Corey L (1997) Frequent detection of Kaposi's sarcoma-associated herpesvirus (human herpesvirus 8) DNA in saliva of human immunodeficiency virus-infected men: clinical and immunologic correlates. *J Infect Dis* **176**: 94–102

Lennette ET, Blackbourn DJ and Levy JA (1996) Antibodies to human herpesvirus type 8 in the general population and in Kaposi's sarcoma patients. *Lancet* **348**: 858–861

Martin JN, Ganem DE, Osmond DH, Page-Shafer KA, Macrae D and Kedes DH (1998) Sexual transmission and the natural history of human herpesvirus 8 infection. *N Engl J Med* **338**: 948–954

Mayama S, Cuevas LE, Sheldon J, Omar OH, Smith DH, Okong P, Silvel B, Hart CA and Schulz TF (1998) Prevalence and transmission of Kaposi's sarcoma-associated herpesvirus (human herpesvirus 8) in Ugandan children and adolescents. *Int J Cancer* **77**: 817–820

Melbye M, Cook PM, Hjalgrim H, Begtrup K, Simpson GR, Biggar RJ, Ebbesen P and Schulz TF (1998) Risk factors for Kaposi's-sarcoma-associated herpesvirus (KSHV/HHV-8) seropositivity in a cohort of homosexual men, 1981–1996. *Int J Cancer* **77**: 543–548

Olsen SJ, Chang Y, Moore PS, Biggar RJ and Melbye M (1998) Increasing Kaposi's sarcoma-associated herpesvirus seroprevalence with age in a highly Kaposi's sarcoma endemic region, Zambia in 1985. *Aids*, **12**: 1921–1925

Rabkin CS, Schulz TF, Whitby D, Lennette ET, Magpantay LI, Chatlynne L and Biggar RJ (1998) Interassay correlation of human herpesvirus 8 serologic tests. HHV-8 Interlaboratory Collaborative Group. *J Infect Dis* **178**: 304–309

Renwick N, Halaby T, Weverling GJ, Dukers NH, Simpson GR, Coutinho RA, Lange JM, Schulz TF and Goudsmit J (1998) Seroconversion for human herpesvirus 8 during HIV infection is highly predictive of Kaposi's sarcoma. *Aids* **12**: 2481–2488

Rezza G, Lennette ET, Giuliani M, Pezzotti P, Caprilli F, Monini P, Butto S, Lodi G, Di Carlo A, Levy JA and Ensoli B (1998) Prevalence and determinants of anti-lytic and anti-latent antibodies to human herpesvirus-8 among Italian individuals at risk of sexually and parenterally transmitted infections. *Int J Cancer* **77**: 361–365

Simpson GR, Schulz TF, Whitby D, Cook PM, Boshoff C, Rainbow L, Howard MR, Gao SJ, Bohenzky RA, Simmonds P, Lee C, de Ruiter A, Hatzakis A, Tedder RS, Weller IV, Weiss RA and Moore PS (1996) Prevalence of Kaposi's sarcoma associated herpesvirus infection measured by antibodies to recombinant capsid protein and latent immunofluorescence antigen. *Lancet* **348**: 1133–1138

Sitas F, Carrara H, Beral V, Newton R, Reeves G, Bull D, Jentsch U, Pacella-Norman R, Bourboulia D, Whitby D, Boshoff C and Weiss R (1999) The seroepidemiology of HHV-8/KSHV in a large population of black cancer patients in South Africa. *N Engl J Med* **340**: 1863–1871

Smith N, Sabin C, Gopal R, Bourboulia D, Labbet W, Boshoff C, Barlow D, Band B, Peters B, de Ruiter A, Brown D, Weiss R, JM B and Whitby D (1999) Serological evidence of human herpesvirus 8 (HHV-8) transmission by homosexual but not heterosexual sex. *J Infect Dis* **180**: 600–606

Vieira J, Huang ML, Koelle DM and Corey L (1997) Transmissible Kaposi's sarcoma-associated herpesvirus (human herpesvirus 8) in saliva of men with a history of Kaposi's sarcoma. *J Virol* **71**: 7083–7087

Whitby D and Boshoff C (1998) Kaposi's sarcoma herpesvirus as a new paradigm for virus-induced oncogenesis. *Curr Opin Oncol* **10**: 405–412

Whitby D, Luppi M, Barozzi P, Boshoff C, Weiss RA and Torelli G (1998) Human herpesvirus 8 seroprevalence in blood donors and lymphoma patients from different regions of Italy. *J Natl Cancer Inst* **90**: 395–397

Ziegler JL and Katongole-Mbidde E (1996) Kaposi's sarcoma in childhood: an analysis of 100 cases from Uganda and relationship to HIV infection. *Int J Cancer* **65**: 200–203

 *© 2000 Cancer Research Campaign*

